# Impact of night shift work on women's fertility, pregnancy and menopause

**DOI:** 10.3389/frsle.2025.1545258

**Published:** 2025-04-10

**Authors:** Christina Y. Lee, Katherine Moawad, Grace W. Pien

**Affiliations:** ^1^Department of Sleep Medicine, ChristianaCare Health System, Newark, DE, United States; ^2^Division of Sleep Medicine, Department of Medicine, University of Pennsylvania Perelman School of Medicine, Philadelphia, PA, United States

**Keywords:** shift work, pregnancy outcomes, birth outcome, working hours, fertility, fecundability, menopause

## Abstract

A growing body of literature examines how shift work affects different aspects of women's reproductive cycles, ranging from fertility to pregnancy to menopause. This review summarizes what is known about how shift work affects women's reproductive cycles, with a particular emphasis on fertility, fecundity and the impact of shift work on maternal and fetal outcomes. While the overall impact of night shift work is complex and remains incompletely understood, evidence suggests that shift work adversely impacts reproductive health and pregnancy outcomes.

## 1 Introduction

In industrialized societies, approximately 15–20% of workers are shift workers (Bonnefond et al., 2004). While shift work does not have a strict definition, night shift is often defined as working ≥3 h, between the hours of 2,200 to 0,600 (Hammer et al., 2018; Specht et al., 2019; Kader et al., 2022). The definition of rotating shift work is also variable, with some papers describing alternating between day, evening, and night shifts within 1 week, vs. shifts alternating over longer intervals (Infante-Rivard et al., 1993; Xu et al., 1994). According to the U.S. Bureau of Labor Statistics, in 2017–2018, 16% of workers usually worked a non-daytime schedule, including 4% who worked nights. Females were less likely to work non-daytime schedules than males (15.2% compared to 17.6%), but a larger proportion of females worked the night shift (3.9% compared to 3.3% of males) (Bureau of Labor Statistics U.S. Department of Labor, 2019).

Industries that necessitate night shift work include healthcare, hospitality, transportation, aviation, food services, and first responders (Working Group, 2020). As healthcare is well known to employ shift workers for patient care and employs over 22 million adult Americans (Centers for Disease Control and Prevention, 2024), many people may experience shift work at some point of their training or career (Blytt et al., 2022; Jackson and Moreton, 2013). Because of this, many studies (Kader et al., 2022; Whelan et al., 2007; Begtrup et al., 2019; Davari et al., 2018; Begtrup et al., 2023; Agarwal et al., 2023; Nurminen, 1989) examining the impact of shift work focus on or include a large proportion of healthcare workers (Ganesan et al., 2019). Nocturnal shift work can disrupt lifestyle, mental health, and physical health; and can provoke irregular sleep wake cycles and circadian rhythm disturbances, leading to hormonal imbalance (Antunes et al., 2006; Davis et al., 2012). However, not much is known about how shift work affects different aspects of women's reproductive cycles, ranging from menstruation to fertility to menopause. Understanding the biological effects of night shift work on female reproductive health is important to mitigate potentially unfavorable effects of sleep and circadian rhythm disruption on physiologic processes. The purpose of this narrative review is to examine what is known about how shift work affects women's reproductive cycles throughout their lifespan, with a particular emphasis on fertility, fecundity and the impact of shift work on maternal and fetal outcomes.

## 2 Impact of shiftwork on female fertility

### 2.1 Infertility and fecundity

Female infertility has been defined as the inability to conceive within 12 months for women under 35 years of age or 6 months for women 35 years and older (Practice Committee of the American Society for Reproductive Medicine, 2013). Reasons for female infertility include menstrual irregularity, ovulatory dysfunction, tubal dysfunction, endometriosis, uterine dysfunction, and unexplained factors (Carson and Kallen, 2021). Additional factors influencing female fertility include maternal age, medical comorbidities, and environmental factors (Carson and Kallen, 2021). Fecundity is the biological capacity for humans to reproduce. As there is no biomarker to assess population fecundity, it is assessed indirectly using various individual (e.g., ovulation) or couple-related (e.g., time to pregnancy) endpoints (Smarr et al., 2017). Fertility and fecundity rates have been declining in recent years (Bhattacharjee et al., 2024; Okhovati et al., 2015; Gliozheni et al., 2020). As the number of female shift workers has increased, this has led to speculation about whether shift work adversely affects fertility and fecundity.

### 2.2 Fertility and circadian rhythm

Most reproductive hormones essential for female fertility, including luteinizing hormone (LH), follicle stimulating hormone (FSH), estrogen and testosterone, demonstrate circadian rhythmicity (Gamble et al., 2013; Sciarra et al., 2020). Regulation of the estrus cycle and LH surge are regulated by circadian clock genes (reviewed in detail in reference (Carson and Kallen, 2021). Dyssynchronous exposure to light caused by shiftwork can lead to loss of rhythmicity in the suprachiasmatic nucleus, disrupting normal pulsatile sexual hormone release and altering overall fertility (Sciarra et al., 2020). Disruption of clock genes has been associated with lower progesterone levels, irregular estrous cycles, fewer ovarian follicles and higher rates of pregnancy failure in animal models (Zheng et al., 2019; Boden et al., 2010; Liu et al., 2014). Most studies exploring the relationship between reproduction and circadian rhythm have been performed in rodent models investigating clock knockout genes, rather than experiments meant to simulate shift work (Rahman et al., 2019). In humans, night and rotating shift work can induce some degree of desynchronization among different hormonal systems due to the effects of sleep and circadian disruption on normal hormonal secretory patterns (Gamble et al., 2013; Sciarra et al., 2020; Fernandez et al., 2020; Kerdelhué et al., 2002).

Additionally, melatonin interacts with gonadotropins, potentially enhancing the LH surge (Fernandez et al., 2020; Zhu et al., 2003; Wiegand and Terasawa, 1982). Normal melatonin secretion begins in the evening, peaks in the early morning hours and then declines to low levels by midday (Burgess and Fogg, 2008). Shift workers remain entrained to this rhythm despite misalignment with the timing of the sleep/wake cycle. However, they experience reductions in melatonin levels due to sleep deprivation and nocturnal light exposure, which can contribute to hypothalamic pituitary axis deregulation (Bracci et al., 2013). Female nurses working forward rotating shifts have been observed to have reduced circadian variation in skin temperature compared to nurses working daytime shifts (Bracci et al., 2016). Lower levels of intrafollicular melatonin have been observed among women with unexplained infertility, though whether this is related to sleep or circadian disruption is unknown (Espino et al., 2019). Circadian rhythm regulation is important not only for maintaining the intricate and time-sensitive pathways of reproductive hormone synthesis and secretion, but also for facilitating reproductive processes including folliculogenesis, ovulation, fertilization, and implantation (Zheng et al., 2019; Beroukhim et al., 2022; Sen and Hoffmann, 2020; Kalantaridou et al., 2004; Wiggins and Legge, 2016; Cunningham et al., 2018). Overall, while data have not been replicated in humans, investigations in animal models illustrate how circadian rhythm disruption caused by nightshift work can potentially adversely affect fertility outcomes and female reproductive health.

### 2.3 Menstrual irregularities and sleep

A normal menstrual cycle lasts between 21 and 35 days, with a median duration of 28 days and bleeding typically lasting from 2 to 6 days (Cunningham et al., 2018; Stocker et al., 2014). A meta-analysis of studies investigating various sleep and fertility parameters concluded that disrupted sleep is linked to a 46% higher likelihood of experiencing menstrual irregularities (Auger et al., 2021). Irregular circadian rhythms have been linked to menstrual cycle dysfunction, with sleep disruption being one of the key physiological factors contributing to this relationship (Stocker et al., 2014; Auger et al., 2021). In a study investigating the causes of subfertility, night shift workers showed 30-40% higher likelihoods of experiencing menstrual irregularities (OR = 1.42, 95% CI 1.05–1.91) and diagnosis of endometriosis (OR = 1.34, 95% CI 1.00–1.80) compared to day shift workers (Fernandez et al., 2020). Consistent with previous research, menstrual irregularities have been reported at higher prevalence among nurses working at night (Lawson et al., 2015; Mínguez-Alarcón et al., 2017). An overview of recent work in this area is beyond the scope of this manuscript but has been recently undertaken by Beroukhim et al. (2022).

These studies underscore the importance of considering occupational factors when evaluating fertility outcomes and highlight the need for further research and workplace interventions to protect reproductive health in women. While there is a growing body of research on the relationship between sleep disturbances and menstrual irregularities, there is still a paucity of comprehensive studies that fully explore the mechanisms, causality, and broader implications of sleep disruption on menstrual health. While menstrual irregularities are widely acknowledged to affect fertility, this review will focus on data related to assisted reproductive technology and fecundity in night shift work.

### 2.4 Night shift work, fertility treatment, and fecundity

A number of retrospective and prospective studies have identified adverse relationships between shift work and fertility indicators (Zhu et al., 2003; Bisanti et al., 1996; Ahlborg et al., 1996). Conversely, other studies, including prospective (Gaskins et al., 2015), retrospective (Zhu et al., 2003), and cross-sectional research (Tuntiseranee et al., 1998), found little association between shift work and female fertility (Fernandez et al., 2020; Sponholtz et al., 2021). Understanding the circumstances under which night shift work adversely impacts female fertility is essential to identifying strategies for mitigating risks for women planning to conceive while working non-standard hours.

A retrospective analysis of 128,852 primiparous women from Australia (Fernandez et al., 2020) examined whether women who worked night shift were more likely than day workers to require fertility treatment to conceive a first birth. Among women 35 years old and younger, night shift workers (*N* = 11,000, 8.5%) were significantly more likely to require fertility treatment to conceive first birth (OR = 1.40, 95% CI 1.19–1.64) compared to day workers (Fernandez et al., 2020). The greater use of fertility treatment among night shift workers was magnified when they were compared with all primiparous women, including those not in paid employment (Fernandez et al., 2020). Furthermore, increased use of fertility treatment among night shift workers compared to other participants persisted when nurses, who formed the largest proportion of night shift workers, were excluded from the analyses.

Higher rates of night shift work have been observed in African American women, with limited evidence suggesting lower fertility rates in this population compared to non-Hispanic white women (Sponholtz et al., 2021; Bitler and Schmidt, 2006). A recent analysis assessed the association between night shift work and time to pregnancy in the Black Women's Health Study (BWHS), a large prospective cohort study of more than 50,000 self-identified African American women, conducted using questionnaires (Sponholtz et al., 2021). The study assessed fecundability, defined as the probability of achieving pregnancy within a single menstrual cycle among couples who were actively trying to conceive (Sponholtz et al., 2021). Time to pregnancy was defined as the reported number of months between the beginning of the pregnancy attempt and conception. Fecundability among African American night shift workers (*n* = 560) aged 30–45 years old who reported ever working night shifts in years prior was 20% lower compared with those who never reported night shift work after adjusting for potential confounders (Sponholtz et al., 2021). Further analyses showed reduced fecundability was concentrated among women aged ≥35 years who had reported ever working night shifts; and the presence of a dose response relationship between fecundability and the frequency and duration of night shift work (Sponholtz et al., 2021). Overall, a history of night shift work was associated with reduced fecundability among older reproductive-aged African American women who were actively trying to conceive.

The relationship between female sleep patterns, shift work and fecundability has also been evaluated in a prospective online pre pregnancy cohort of mostly white North American women (*n* = 6,873) (Willis et al., 2019). In this study, shift work (rotating shifts or night shifts) was not associated with a significant reduction in fecundity; instead, an 11% reduction in fecundity was observed among those reporting short sleep of less than 6 h per night, although this was not statistically significant. The study concluded that sleep patterns, particularly trouble sleeping and very short sleep, may influence fertility, though the effects are modest. The overall extent to which female fertility is influenced by shift work and sleep patterns remains unsettled.

## 3 Impact of shiftwork on pregnancy and maternal fetal outcomes

Multiple studies have looked at the effects of night shift work and/or rotating shift work on pregnancy compared to day shift work (Hammer et al., 2018; Specht et al., 2019; Kader et al., 2022; Infante-Rivard et al., 1993; Xu et al., 1994; Cunningham et al., 2018; Stocker et al., 2014; Auger et al., 2021; Lawson et al., 2015; Mínguez-Alarcón et al., 2017; Bisanti et al., 1996; Ahlborg et al., 1996; Gaskins et al., 2015; Tuntiseranee et al., 1998; Sponholtz et al., 2021; Bitler and Schmidt, 2006; Willis et al., 2019; Axelsson et al., 1989, 1996; Zhu et al., 2004). However, only a few studies have included non-working women as a comparison group (Chang et al., 2010; Suzumori et al., 2020; Lee et al., 2024). The most heavily studied maternal outcomes include hypertensive disorders of pregnancy (gestational hypertension, pre-eclampsia, eclampsia) and gestational diabetes (Hammer et al., 2018; Davari et al., 2018; Agarwal et al., 2023; Nurminen, 1989; Larson et al., 2022; Wallace et al., 2023; Chang et al., 2010; Suzumori et al., 2020). Among fetal outcomes, the most data exist regarding the relationship between shift work and preterm birth, small for gestational age (SGA), low birth weight (LBW), and spontaneous abortion (Specht et al., 2019; Kader et al., 2022; Infante-Rivard et al., 1993; Xu et al., 1994; Whelan et al., 2007; Begtrup et al., 2019; Davari et al., 2018; Begtrup et al., 2023; Axelsson et al., 1989, 1996; Zhu et al., 2004; Bonzini et al., 2009; Moćkun-Pietrzak et al., 2022; Larson et al., 2022; Wallace et al., 2023; Bond et al., 2024; Izadi et al., 2024; Lee et al., 2024).

### 3.1 Gestational diabetes

Few papers have examined the relationship between shiftwork and gestational diabetes (GDM) (Larson et al., 2022; Wallace et al., 2023; Suzumori et al., 2020). Overall, they suggest an association exists between night shift work and gestational diabetes.

Wallace et al. (2023) used data from the nuMoM2b study, an NIH-funded, prospective cohort of pregnant participants from eight large US hospitals, to examine the relationship between shift work and gestational diabetes. Among 5,191 participants without prior diabetes or chronic hypertension who reported first-trimester work shift history and had pregnancy outcome data, 11.9% reported afternoon or night shift work. They described an approximately 75% increased odds of developing gestational diabetes among afternoon and night shift workers compared to day workers after adjusting for potential confounders. Interestingly, participants who worked irregular or rotating shifts did not have a greater risk of GDM.

Over 900 individuals from the nuMoM2b cohort also wore actigraphy watches during the second trimester to document sleep-wake patterns (Wallace et al., 2023). Investigators determined that night shift workers had much greater variability in their sleep timing compared to afternoon workers, suggesting that a substantial portion of the association between shift work and GDM is likely mediated by sleep timing variability.

Suzumori et al. (2020) examined the relationship between night shift work and gestational diabetes in a large prospective nationwide cohort in Japan (n=104,102), comparing different groups of working women to non-workers, who comprised 47.2% of the cohort. Women who worked ≥46 h/week without night shifts in the second/third trimesters had a significantly lower risk for GDM than non-workers. Modestly lower GDM risks for self-identified night shift workers compared to non-workers were not statistically significant. However, this study did not directly compare GDM risk between night and day shift workers.

### 3.2 Hypertensive disorders of pregnancy

Multiple studies have examined the relationship between shift work and hypertensive disorders of pregnancy (gestational hypertension, pre-eclampsia, and eclampsia) (Hammer et al., 2018; Davari et al., 2018; Agarwal et al., 2023; Nurminen, 1989; Larson et al., 2022; Wallace et al., 2023; Chang et al., 2010; Suzumori et al., 2020). While some studies did not observe higher risk of adverse blood pressure outcomes associated with shift work (Davari et al., 2018; Agarwal et al., 2023; Chang et al., 2010), others showed an increased risk with a dose response relationship (Hammer et al., 2018; Suzumori et al., 2020).

An interesting study performed by Hammer et al. (2018) investigated the relationship between shift work, shift recovery time, and hypertensive outcomes in pregnancy. Shifts lasted ≥3 h and were categorized as day, evening, night or early morning shifts. After adjusting for confounding factors, women who worked 4 or more consecutive night shifts at least once in the first 20 weeks of pregnancy demonstrated a greater risk of hypertensive disorders of pregnancy, compared to night workers who did not work consecutive night shifts. However, the risk was not significantly increased among the group with the most intensive exposure, women with at least 5 quick returns after a night shift (i.e., recovery periods of <28 h after a night shift). Among women with obesity (BMI ≥30 kg/m^2^) who worked 4 or more consecutive night shifts, the risk for hypertensive disorders of pregnancy was higher compared to day shift workersMore generally, however, night workers did not demonstrate significantly greater risk for hypertensive disorders of pregnancy compared to day workers. The authors suggested that the effect of night work on the risk of hypertensive disorders of pregnancy was likely to be related to the way night shift was organized, rather than simply the performance of night shift work.

### 3.3 Small for gestational age, low birth weight

Several studies have looked at the relationship between shift work and SGA births, defined as weight at birth less than 10th percentile for gestational age, with conflicting results (Davari et al., 2018; Begtrup et al., 2023; Zhu et al., 2004; Bonzini et al., 2009; Lee et al., 2024). Zhu et al. (2004) interviewed a large, nationally representative cohort of Danish women (*n* = 54,954) at two separate time points during pregnancy for information about shift work. Birth data was obtained from the National Birth Register in the Danish National Birth Cohort. Those who performed rotating shift work (with and without nights) had a small increase in risk for SGA compared to daytime workers that was of borderline significance (OR 1.09, 95% CI 1.00–1.18).

Begtrup et al. (2023) combined payroll data from the Danish Working Hour Database with medical data extracted from the Danish Medical Birth Registry. This study was distinguished by the high proportion of night workers (61% of total pregnancies). Most night workers were nurses and physicians (81 vs. 23% of day workers). Night shifts were defined as ≥3 h of work between 2,300 and 0600 h, day shifts as ≥3 h between 0600 and 2,100 h. The authors found that night work, compared with day work, was not associated with increased risk for SGA birth.

Using data from more than 5,200 pregnant women in the Korean Children's Environment Health Study (Ko-CHENS), a South Korean nationwide prospective birth cohort, Lee et al. found no significant differences in the adjusted odds ratios of SGA between day workers (no shift work), day shift workers (shift work without night shifts), and night workers (shift work with night shifts) (Lee et al., 2024). There were very few night workers (*n* = 34) compared to day shift workers (*n* = 286) and day workers (*n* = 2,924), likely due to national labor restrictions mandating that employers cannot force pregnant women to work between 2,200 to 0600 h. Nevertheless, Lee and colleagues noted a significantly increased risk of SGA for night shift workers compared to non-workers. In smaller studies performed by Bonzini et al. (2009) and Davari et al. (2018), there were no statistically significant associations between night shift work and SGA.

Few studies have examined whether shift work increases the propensity for low birth weight, defined as weight at birth <2,500 g regardless of gestational age (Xu et al., 1994; Davari et al., 2018; Axelsson et al., 1989; Zhu et al., 2004; Izadi et al., 2024). In the earliest of these studies, Axelsson et al. (1989) retrospectively identified all women permanently working nights in one Swedish hospital, and randomly selected a similar group not working nights for comparison. Pregnant women who reported being non-smokers and working irregular hours, night shifts, or rotating shifts had infants with significantly lower birth weights compared to non-smoking women who worked days only. However, there were no differences in the rate of LBW births between groups. A major study limitation was the reliance on participant recall (0 to 30 years prior) of work schedules, duties and habits during pregnancy, as well as infant birthweight.

Xu et al. (1994) focused on birth outcomes among a sample of 1,035 non-smoking women workers in three textile mills in China. Production workers performed forward rotating shifts on an 8 day cycle – morning shift (0600 to 1,400 h for 2 days), evening shift (1,400 to 2,200 h for 2 days), night shift (2,200 to 0600 h for 2 days), followed by 2 days off. Day shift only workers mostly included administrative staff. After adjusting for maternal age, number of live births, multiple occupational factors and coal stove use, the authors found rotating shift work doubled the risk for LBW (OR 2.1, 95% CI 1.1–4.1) compared to day shift work. Like the Axelsson study, this study also relied on participant recall of work duties and birthweight, though the recall period was considerably shorter, given mean maternal age at pregnancy (23.8 years, SD 2.5 years) and study interview (28.7 years).

Contrastingly, more recent analyses performed by Davari et al. (2018) and Izadi et al. (2024) did not show significant relationships between shift work and LBW. Both studies were constrained by numerous methodological limitations.

### 3.4 Preterm birth

A number of studies have looked at the relationship between shift work and preterm birth, defined as delivery at less than 37 weeks gestation, with mixed results (Specht et al., 2019; Kader et al., 2022; Xu et al., 1994; Davari et al., 2018; Bonzini et al., 2009; Larson et al., 2022; Wallace et al., 2023; Lee et al., 2024). In the previously described prospective nuMoM2b cohort (Wallace et al., 2023) and largely Latinx Harris County cohort (Larson et al., 2022), there were no significant associations between night shift work and preterm birth. Bonzini et al. (2009) incorporated information about job duties such as lifting and standing in their assessment of work schedules and also did not find an association between long working hours (≥40 h/week), lifting and standing, and night shift work and preterm birth. The Ko-CHENS study (Lee et al., 2024) did not find a link between preterm birth and night shift work compared to non-workers, but as noted previously, was limited by the very small number of night workers. In contrast, Xu et al. (1994) found that among textile workers, there was an adjusted OR of 2.0 for preterm birth among rotating shift workers compared to day workers.

Two recent studies have evaluated trimester differences in the potential impact of shift work on preterm birth (Specht et al., 2019; Kader et al., 2022). Specht et al. (2019) analyzed data from 16,501 pregnant women enrolled in the Danish Working Hour Database and linked to the Danish Medical Birth Registry. They did not find significant differences in the odds for preterm birth between night and day shift workers during the first or second trimesters regardless of number, duration, or quick returns of night shifts. However, a modestly increased odds of preterm birth was observed among women who changed from night shifts in the first trimester to day shifts in the second trimester, compared to women working night shifts in both trimesters. This could be partially explained by the healthy worker survivor effect, wherein unhealthy or ill workers change from night to day shift.

Within a large cohort of Swedish healthcare professionals, Kader et al. (2022) compared associations between night shift work and preterm labor to day workers across trimesters of pregnancy (*n* = 4,970). After adjusting for confounders, the investigators observed a modestly increased risk of preterm birth among women who frequently worked night shifts in the first trimester (>25 times) and those who had worked ≥3 consecutive night shifts, compared to women who did not do night work and instead worked day or afternoon shifts during their first trimester. Frequently working ≥3 consecutive nights (>8 times) in the first trimester was associated with a three-fold risk for preterm birth compared to working ≥3 consecutive nights 1–4 times in the first trimester. Likewise, frequent quick returns (>18) from night shifts (recovery period of <28 h after a night shift) in the first trimester was associated with a greater than twofold risk for preterm labor, compared to a first trimester night shift schedule with 1–8 quick returns. Similar increases in risk for preterm birth were observed for first-time mothers performing night shift work in the second and third trimesters. Overall, it appeared that there was a dose dependent response between the length and frequency of night shifts and the risk for preterm birth.

### 3.5 Pregnancy loss, abortion, miscarriage

The handful of papers that have examined the relationship between shiftwork and outcomes of pregnancy loss and spontaneous abortion/miscarriage suggest night shift work may increase risk for these outcomes (Infante-Rivard et al., 1993; Whelan et al., 2007; Begtrup et al., 2019; Davari et al., 2018; Axelsson et al., 1989, 1996; Moćkun-Pietrzak et al., 2022; Bond et al., 2024; Izadi et al., 2024). Of note, definitions for spontaneous abortion or miscarriage have varied depending upon the geographic region, as there is no universal consensus as to what gestational age distinguishes miscarriage from stillbirth. The majority of papers described miscarriage as pregnancy loss at less than 20 weeks gestational age. However, one paper defined miscarriage as pregnancy loss up to 22 weeks (Begtrup et al., 2019) and another paper up to 29 weeks (Axelsson et al., 1996).

Infante-Rivard et al. (1993) compared work schedules of pregnant women presenting to a single university-affiliated Canadian hospital who had experienced pregnancy loss (*n* = 331) to those who had not (*n* = 993), matching them by gestational age. Work schedules were divided into fixed days, fixed evenings (1,500 or 1,600 to 2,300 or 0000 h), fixed nights, or rotating shifts. Overall, 74% of pregnancy losses occurred before a gestational age of 17 weeks, 13% between 17 to 20 weeks, and 13% after 20 weeks. After adjusting for age, education level, presence of children at home, coffee intake, and uterine anatomical abnormality, they found that working a fixed evening schedule was associated with an approximately fourfold increased risk for pregnancy loss compared to day shift workers. No significant relationship between night shift work and pregnancy loss was observed. However, fewer than 2% of cases or controls reported regular night shift work.

Within the Nurses' Health Study II cohort, Whelan et al. (2007) collected information on nurses' work schedules and pregnancy outcomes within the prior 8 years via questionnaires. Night shift was defined as most work hours occurring between 0000 and 0800 hours. Analyses demonstrated a 60% increased risk of spontaneous abortion (involuntary pregnancy loss prior to 20 weeks gestation) among women working nights only during the first trimester, compared to women working days only. Working a rotating schedule, with or without nights, was not associated with increased risk for spontaneous abortion.

Begtrup et al. (2019) investigated a cohort of 22,744 pregnant women in the Danish Working Hour Database linked with registry data on hospital admissions to identify miscarriages. Night shift was defined as working at least 3 h between 0000 and 0500 hours. Miscarriages were defined as pregnancy loss between 4 and 22 weeks gestation. The majority of the cohort were hospital-based employees, and 44% of participants were exposed to night shift work during pregnancy weeks 3–21. Overall, participants who had worked night shifts had a dose dependent risk of modestly higher numbers of miscarriage compared with those who had not worked any night shifts during pregnancy weeks 3–21. This risk was the greatest among women working 26 or more night shifts; however, the number of pregnancy losses were few (*n* = 8). There was a small but significant increased risk for miscarriage for women at 9 weeks gestation and above who worked at least 2 night shifts the prior week compared to women who did not work night shifts the week prior, which was heightened among women aged 26–30.

Moćkun-Pietrzak et al. (2022) distributed questionnaires containing questions about reproductive health to a group of Polish midwives (*n* = 520). reported While more midwives who worked some overnight shifts reported miscarriages compared with those working days only, the authors did not report whether these differences were statistically significant.

Interestingly, Bond et al. (2024) prospectively examined the relationship between shiftwork and miscarriage among North American couples who were recruited while trying to conceive. All study communications were online through self report. These women (*n* = 17,083) were followed through pregnancy, or until cessation of pregnancy attempt, study withdrawal, or 12 months without a pregnancy. Working at night was defined as working at least some time between 0000 and 0200 h, and rotating shifts were defined as working hours that shifted daily, weekly, or monthly. In adjusted analyses, women who performed night work (*n* = 1,142) had modestly but significantly higher rates of spontaneous abortion compared to those who had not performed night or rotating shift work. Female participants who reported a pregnancy could invite their male partners to participate (*n* = 2,602); 356 male participants reported non-daytime work. A modest but statistically significant increased risk of spontaneous abortion was observed among couples in which the male partner reported non-daytime work (evening, night, or rotating shift work), compared with couples who did not report non-daytime work.

Overall, shift work may adversely affect pregnancy outcomes. Many studies looking at birth outcomes in pregnant shift workers enrolled healthcare workers such as nurses and midwives, whereas few studies examined population-wide outcomes. Likely reasons for studies focusing on healthcare workers include: (1) night shifts are more frequent in this population due to the “nature of the job” (Beers, 2000); (2) working hours can be documented through hospital time logs or payroll data that can be analyzed retroactively, and these records may be easily linked to health and birth data to reduce recall bias; and (3) healthcare professionals may be more sensitized to health events and therefore events may be more adequately recalled on self-reported questionnaires (Whelan et al., 2007).

Limitations to this data include recall bias in studies performed using questionnaires; often, work schedules and/or medical outcomes were unable to be verified. Many studies noted possible healthy worker effect, as women who worked night shift may have been healthier than those who chose to work day shift; alternatively, women with health concerns may have chosen to switch from night shift to day shift. There also was no information regarding if these women were able to take naps during their night shift work. Confounding factors may not have been measured in some studies, including work exposures such as positioning (seated vs. standing, lifting, etc.). Given that many studies were retrospective, it is difficult to determine true causation due to shift work despite looking at confounders.

## 4 Impact of shiftwork on menopause

Very little data exists regarding the potential impact of shift work on menopause timing, vasomotor symptoms and other aspects of menopause. To date, we are aware of only one cross-sectional study examining the relationship between shift work and vasomotor symptoms (Sawamoto et al., 2024). Sawamoto et al. assessed responses among female company workers aged 35 years or older (*n* = 685) to an internet survey regarding the frequency of shift work per week (categorized as almost none, 1 to 3 days, or 4 days or more) and the severity of menopausal symptoms (no/few, mild, or moderate to severe). When looking at the subset of women who experienced moderate to severe menopausal symptoms (*n* = 213), respondents working 1 to 3 night shifts per week were significantly more likely to report severe menopausal symptoms compared to women who worked almost no night shifts (OR 1.93, 95% CI 1.05–3.55).

Only two longitudinal studies have examined whether shift work affects the onset of menopause (Khan et al., 2022; Stock et al., 2019). Stock et al. (2019) first examined whether shift work affects menopause onset in a population of 80,840 female nurses aged 25 to 42 at cohort inception in 1989 and followed to 2013. Shift work was defined as working at least 3 nights per month with day or evening shifts otherwise. By study completion in 2013, 34% of women reported menopause including 9% of women exposed to night shift work who reported menopause occurrence before age 45. In multivariable models adjusted for different aspects of prior shift work, the authors observed a moderate increase in odds of earlier onset menopause for women who worked ≥10 months of rotating night shift work in the previous 2 year interval, compared to women who did not work rotating night shift work. Among women younger than 45 years, prolonged exposure to night shift work was associated with a 22% increased risk of earlier menopause compared to no rotating night shift exposure.

In contrast, Khan et al. (2022) examined data from 3,688 premenopausal women enrolled in a longitudinal cohort of adults representing the general population of Canada. They were followed prospectively for 3 years and asked to describe current and past working schedules (daytime work, night shift, rotating shift) at baseline, and whether they had experienced menopause, defined as cessation of menstrual periods for at least 1 year. Approximately 20% reported exposure to night or rotating shift work. Women with any exposure to night or rotating shifts were more likely to have later onset of menopause (median age 55) compared to daytime workers (median age 54). Contradictory findings of later vs. earlier menopause observed among current night shift workers and current rotating shift workers compared to daytime workers were not statistically significant.

It is important to note that there may be differences between these 2 study populations (i.e., a nurse population and the general population) such as varying levels of stress that can affect hormonal balances, which in turn may affect menopause. Furthermore, the Canadian cohort was asked to recall their history of shift work over a much longer duration, potentially contributing to misclassification bias. At present, given these limited but conflicting studies, there is insufficient data to determine whether shift work affects the timing of menopause.

## 5 Conclusion

A growing body of evidence supports the impact of shift work, particularly night shifts, on female fertility and maternal fetal health. Nevertheless, the overall impact of these disruptions is complex and remains incompletely understood. The widespread use of assisted reproductive technology limits the ability to establish causality between night shift work and infertility. Additionally, not all women have access to assisted reproductive technology which may not fully represent all infertile women in study cohorts.

After pregnancy is achieved, night shift work may adversely affect pregnancy outcomes. Overall, night shift workers may be at increased risk for gestational diabetes (Larson et al., 2022; Wallace et al., 2023; Suzumori et al., 2020), hypertensive disorders of pregnancy (Hammer et al., 2018; Suzumori et al., 2020), preterm birth (Specht et al., 2019; Kader et al., 2022; Xu et al., 1994), and pregnancy loss (Whelan et al., 2007; Begtrup et al., 2019; Axelsson et al., 1996; Moćkun-Pietrzak et al., 2022; Bond et al., 2024). However, insufficient data exist to predict the effects of frequent night shifts in different trimesters. Several papers suggest a mildly increased risk of preterm birth with long or frequent night shifts (Kader et al., 2022) and mild to moderately increased risk of GDM with presence of any night work (Wallace et al., 2023; Suzumori et al., 2020) during all three trimesters. The data point toward a modestly higher risk of developing hypertensive disorders of pregnancy with a higher number of consecutive night shifts or with more quick returns (Hammer et al., 2018; Suzumori et al., 2020). When looking at pregnancy loss, women working nights appear to have a small increased risk for miscarriage or pregnancy loss within the first trimester in a dose-dependent fashion (Whelan et al., 2007; Begtrup et al., 2019; Axelsson et al., 1996; Moćkun-Pietrzak et al., 2022; Bond et al., 2024).

Clinicians should address sleep disturbances, consider occupational factors in fertility assessments, and provide individualized guidance for women planning to conceive and who become pregnant. Clinicians can play an important role in advocating for workplace interventions that promote better sleep and reduce the potential reproductive risks associated with shift work, including flexible scheduling and sleep-friendly work environments.

Future directions investigating night shift work, fertility and pregnancy outcomes should seek to prospectively define and analyze the relative impacts of interrelated factors including circadian disruption, sleep restriction and sleep disturbance. The effects of external stressors and the role of support systems to identify behavioral and psychological factors that may alter fertility also require further study. Trimester-specific research can provide insight as to when women working night shifts may be the most vulnerable to adverse outcomes and also identify ways to reduce the risks of poor outcomes. Wearable devices may also offer insights by providing longitudinal data on sleep wake cycles in shift workers.
